# A Unique Desiccated Carbon Dioxide (CO_2_) Formulation of d,d-trans-Cyphenothrin, Mirakn^®^ GX: A Novel Alternative to Indoor Ultra-Low-Volume Spraying

**DOI:** 10.3390/insects17070716

**Published:** 2026-07-10

**Authors:** Suzanna Chiang, Sook-Cheng Pang, Sin-Ying Koou, Mohd Zulkefli Abdol Rahman, Lee-Ching Ng, Cheong-Huat Tan

**Affiliations:** 1 Environmental Health Institute, National Environment Agency, 11 Biopolis Way, #06-05/08, Helios Block, Singapore 138667, Singapore; suzanna_chiang@nea.gov.sg (S.C.); pang_sook_cheng@nea.gov.sg (S.-C.P.); sinying.koou@envu.com (S.-Y.K.); ng_lee_ching@nea.gov.sg (L.-C.N.); 2 Eastern Regional Office, National Environment Agency, 174 Sin Ming Drive, Singapore 575715, Singapore; mohd_zulkefli_abd_rahman@nea.gov.sg; 3 School of Biological Sciences, Nanyang Technological University, 50 Nanyang Avenue, Singapore 639798, Singapore; 4 Saw Swee Hock School of Public Health, National University of Singapore, 12 Science Drive 2, #10-01, Singapore 117549, Singapore

**Keywords:** desiccated carbon dioxide, Mirakn^®^ GX, ultra-low-volume spraying, *Aedes*, *Culex*, Singapore

## Abstract

In Singapore, indoor space spraying, such as ultra-low-volume (ULV) treatment, is used to control adult mosquitoes during vector-borne disease outbreaks. This study assessed the efficacy of Mirakn^®^ GX, a pyrethroid-based insecticide formulation combining d,d-trans-cyphenothrin with desiccated carbon dioxide as a propellant. The implications of these findings for operational use will be discussed. Mirakn^®^ GX was evaluated against confined field and laboratory strains of *Aedes aegypti*, *Ae. albopictus* and *Culex quinquefasciatus* under semi-field conditions, following World Health Organization (WHO) guidelines. This study was conducted within an occupied residential apartment unit. An additional evaluation focusing on *Ae. aegypti*, designed to mimic operational practice, was performed using a reduced exposure duration of 10 min. At an application rate of 1 g/m^3^ with 60 min exposure, 100% knockdown and 99.79–100% mortality were observed across all three mosquito species. A shortened 10 min exposure also resulted in complete knockdown and mortality in both laboratory and field strains of *Ae. aegypti*, supporting operational feasibility. Under semi-field conditions, the insecticide formulation showed good penetration into enclosed spaces, indicating its potential use for scenarios where timely management of indoor adult mosquitoes is required, including during outbreaks to kill infected mosquitoes.

## Simple Summary

Mosquito-borne diseases such as dengue and Zika continue to pose significant public health challenges in Singapore, and effective indoor mosquito control is needed during outbreaks. Indoor space spraying is commonly used to kill adult mosquitoes, but its effectiveness can be limited if sprays do not reach hidden areas or if mosquitoes become less susceptible to insecticides. This study tested a new spray formulation, Mirakn^®^ GX, which combines a pyrethroid insecticide with carbon dioxide gas to help the spray spread more effectively indoors. The product was tested in a real residential apartment against three mosquito species commonly found in urban Singapore. The results showed that the insecticide achieved 99.79 to 100% mosquito killing, including those in hidden spaces. The product remained effective even when the exposure time was reduced to 10 min, suggesting it can be used with minimal disruption to residents. These findings highlight the potential of this insecticide to improve indoor mosquito control, benefiting both public health programmes and the community by reducing the risk of mosquito-borne diseases.

## 1. Introduction

*Aedes aegypti* (Linnaeus, 1762) is an efficient vector for multiple arboviruses, including dengue and Zika, which have caused recurring outbreaks in Singapore in 2013, 2016, 2020, and 2022 for dengue [1,2,3,4] and in 2016 and 2023 for Zika [5,6,7]. This mosquito species is highly anthropophilic and endophilic, thriving in urban environments and breeding predominantly in artificial containers [8,9]. Its anthropophagic and anthropophilic nature makes it an efficient vector for disease transmission. As a dengue-endemic country with all four dengue serotypes circulating year-round, Singapore remains at continual risk, which presents significant challenges for public health management [10].

To combat mosquito-borne diseases, Singapore undertakes a multi-pronged dengue prevention and control strategy. This comprehensive approach includes case, mosquito and virus surveillance; elimination of breeding habitats; community outreach and education initiatives; and insecticide application during outbreaks to disrupt transmission chains [11]. In addition, Project *Wolbachia*–Singapore complements conventional vector control measures through the release of male *Wolbachia*-infected *Ae. aegypti* mosquitoes to suppress local *Ae. aegypti* populations and reduce the dengue risk [12].

Indoor space spraying (ISS) is one of the commonly applied adult mosquito control measures, particularly in a dengue outbreak. It involves dispersing fine droplets of insecticide within indoor areas to achieve rapid knockdown and mortality of adult mosquitoes, including those that may be infected [13]. However, in Singapore, *Ae. aegypti* populations have been shown to develop resistance to pyrethroid-based insecticides by WHO bioassay tests. This is likely driven by multiple mechanisms, such as enhanced metabolic detoxification and the presence of knockdown resistance (*kdr*) mutations [14,15,16].

New formulation technologies are being developed to enhance the delivery and effectiveness of insecticides against mosquitoes, such as CO_2_-based formulations that could improve aerosol dispersion and permeation. While CO_2_ has long been used in stored-product pest control [17,18,19], its use in mosquito control is a relatively new approach. It is postulated that carbon dioxide may help improve the dispersion and penetration of insecticides, enabling them to reach hidden spaces where mosquitoes often rest.

This study evaluated the adulticidal efficacy of Mirakn^®^ GX, a Type II pyrethroid formulation containing 0.6% *w*/*w* d,d-trans-cyphenothrin, and using CO_2_ as the propellant, against local field strains of *Ae. aegypti*, *Ae. albopictus* (Skuse, 1894), and *Culex quinquefasciatus* (Say, 1823) [20,21,22,23,24,25]. Previous laboratory and field studies in Malaysia, India and the United States have shown that Gokilaht^®^-S 5% EC, with the same active ingredient, achieved more than 80% efficacy against laboratory-reared field strain *Aedes* mosquitoes [26,27]. These findings collectively underscore the promising potential of d,d-trans-cyphenothrin as an effective active ingredient for mosquito control. Hence, evaluating whether novel formulations can maintain efficacy against locally collected mosquito populations is operationally relevant.

## 2. Materials and Methods

### 2.1. Study Site

Singapore is a Southeast-Asian city-state with a tropical climate, a land area of 728.3 km^2^ and an estimated population of 6.11 million [28]. Locally, more than 95% of the resident population reside in high-rise residential apartment buildings (77.4% of the population reside in public housing and 17.7% reside in private apartments [29]).

**Figure 1:**
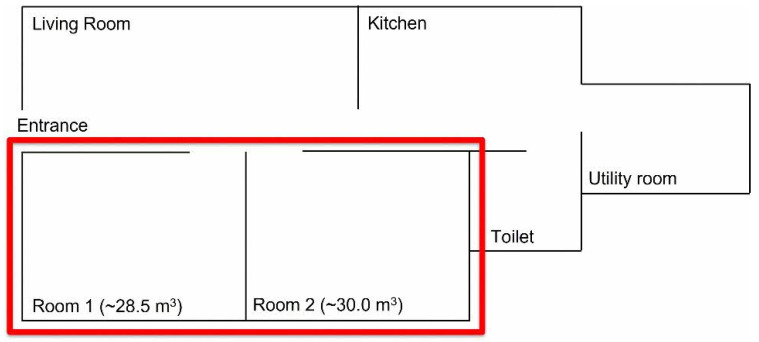
Schematic floorplan of the study site with two test bedrooms (Room 1 and Room 2, boxed red) of similar size.

For this study, an occupied and fully furnished public housing residential apartment unit was selected as a representative of the local household demographic. The unit is located on the 12th floor and has a floor area of 66 m^2^ with a floor-to-ceiling height of 2.5 m. The unit has two adjacent bedrooms of similar sizes, denoted as Room 1 and Room 2 (as depicted in Figure 1).

### 2.2. Mosquito Collection and Rearing

Three common urban mosquito species, *Ae. aegypti*, *Ae. albopictus* and *Cx. quinquefasciatus*, were collected in immature stages (larvae and pupae) from residential and public areas across Singapore during routine inspections by enforcement officers of the National Environment Agency (NEA) [9,20,30,31]. Immature mosquitoes were identified to species based on morphological characteristics [32,33]. Only immatures collected from areas without active dengue, chikungunya and Zika transmission were kept and reared for testing.

Larvae were reared in 37.5 cm × 31.5 cm × 7.0 cm plastic trays containing 1 L of aged water and fed with a slurry made of ground Tetramin^®^ Tropical Flakes (Tetra^®^ Werke GmbH, Melle, Germany). Pupae were placed in 30 cm × 30 cm × 30 cm (L × W × H) cages before emergence into adults. F_0_ adults were allowed to mate randomly, and female mosquitoes were fed with high-health-status animal’s blood (Singapore Health Services Pte Ltd., Singapore) using a Hemotek membrane feeding system (Hemotek Limited, Blackburn, Lancashire, UK) with mouse skin as the membrane. The temperature of the feeding device was set to 37 °C. F_1_ eggs were collected either on Whatman^TM^ No-1 filter papers for *Aedes* mosquitoes (Cytiva, Marlborough, MA, USA) or directly on water and hatched in aged water for *Culex* mosquitoes. Larvae were reared and pupae were allowed to emerge as described above to collect F_2_ eggs. F_2_ adults were used in the study and tested concurrently with their laboratory susceptible strain. Eggs of the laboratory susceptible strain (F_162_ for *Ae. aegypti* (Bora-Bora), F_245_ for *Ae. albopictus* (NEA-EHI), and F_370_ for *Cx. quinquefasciatus* (NEA-EHI)) that were available in the laboratory were reared under the same rearing protocol. All rearing activities were carried out under standard insectary conditions, with an average temperature of 24.4 ± 0.2 °C, relative humidity of 86.6 ± 1.9%, and photoperiod of 12:12 h (light:dark). The rearing protocol was strictly adhered to in order to produce consistent, uniformly sized female adults for the test.

The field-collected mosquito populations used in this study were not subjected to insecticide susceptibility testing prior to evaluation. Consequently, the resistance status of the specific mosquito cohorts tested was not determined.

### 2.3. Insecticide Product

**Figure 2:**
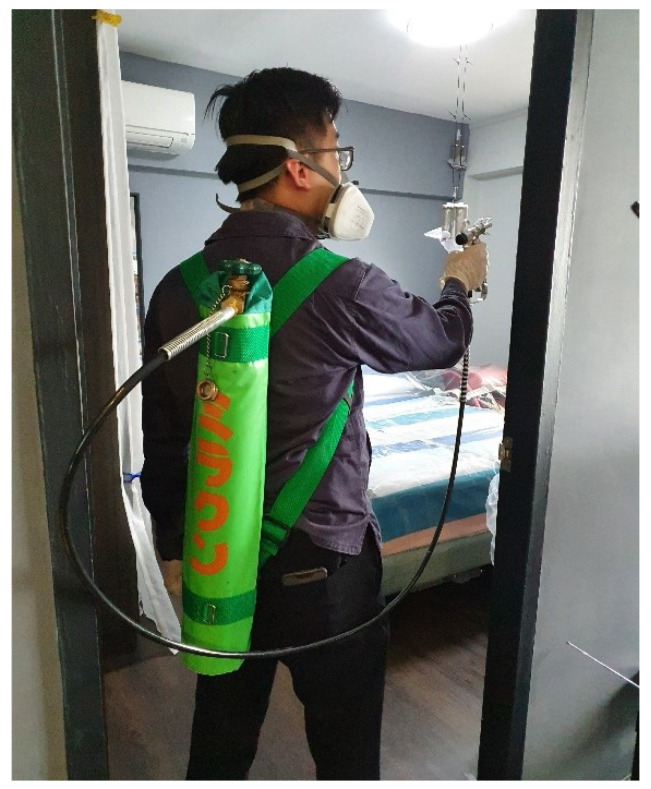
Compressed gas cylinder of Mirakn^®^ GX.

The product evaluated in this study is Mirakn^®^ GX, manufactured by Sumitomo Chemical Co, Ltd., Tokyo, Japan. This formulation consists of a mixture of CO_2_ and d,d-trans-cyphenothrin, supplied in a compressed gas cylinder (as depicted in Figure 2). To ensure precise and consistent application of the recommended dose of 1 g/m^3^, prior to each treatment, the discharge rate of the compressed gas cylinder was verified by weighing it before and after a fixed 5 s calibration spray, ensuring an average discharge rate of 6.5 g/s. A CO_2_-only control treatment was not included because a compressed gas formulation containing CO_2_ without the active ingredient was not available during the study period. Consequently, the present study evaluated the efficacy of the complete commercial formulation and was not designed to determine the independent contribution of CO_2_ to mosquito knockdown or mortality.

### 2.4. Efficacy Test

**Figure 3:**
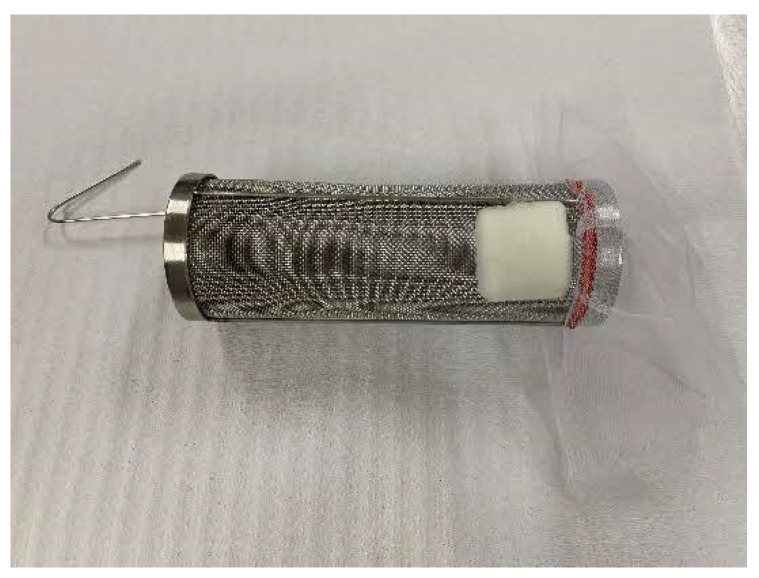
A cylindrical mesh cage with female mosquitoes within.

**Figure 4:**
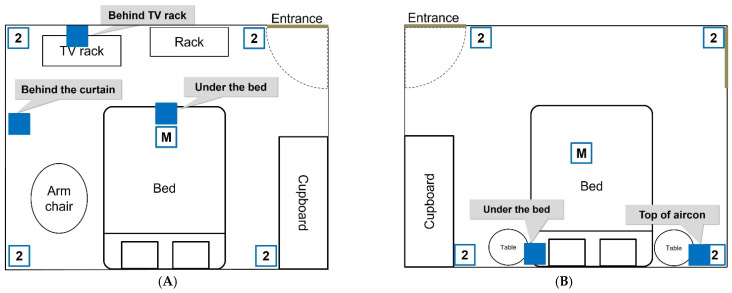
Schematic diagram of Room 1 (**A**) and Room 2 (**B**) showing the mesh cage positions for the 60 min exposure duration. In each mesh cage position, one cage of field strain and one of susceptible strain mosquitoes are placed. Non-filled and filled squares represent exposed and hidden cages, respectively. “2” indicates cages placed 0.25 m below the ceiling and 0.25 m above the floor and “M” indicates cages placed mid-height (1.5 m) at the centre of the room. The dashed arc indicates the door swing path (as depicted in Figure 5).

**Figure 5:**
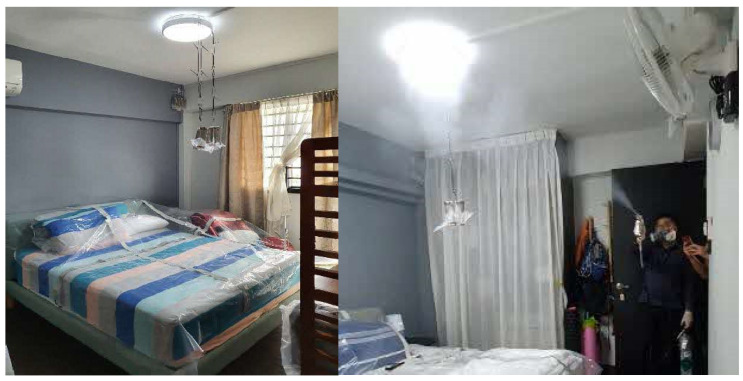
Exposed cages placed 0.25 m below the ceiling and in the middle of Room 1. Actual hidden cages are located behind the curtain and under the bed (**left**). Spraying of Mirakn^®^ GX from the entrance of the room (**right**).

All test procedures were carried out according to the recommendations from the World Health Organization’s (WHO) Guidelines for efficacy testing of insecticides for indoor and outdoor ground-applied space spray applications [34]. Female mosquitoes were transferred into cylindrical mesh cages (5 cm diameter × 15 cm height, as depicted in Figure 3) [35]. Within each test room (volume: 28.5–30.0 m^3^), cages were placed at nine fixed positions: one at mid-height at the centre of the room and four at 0.25 m below the ceiling and 0.25 m above the floor. Two to three additional cages were placed at typical mosquitoes resting sites, such as under the bed [36] (as depicted in Figure 4 and Figure 5). All fans and air-conditioning units were switched off and all windows and doors in the rooms were closed during the test. Temperature and relative humidity were recorded. Cages containing mosquitoes were placed in the room (at the positions stated above) for 60 min and were checked for knockdown or mortality before spraying.

Mirakn^®^ GX was sprayed for 5 s (calculated based on volume of room and average discharge rate) from the entrance of the room (as depicted in Figure 5) from left to right at the recommended application dose of 1 g/m^3^. The room was then kept shut for 60 min (exposure duration) to ensure mosquito exposure to the insecticide. A pad was placed at the bottom of the door to prevent any leakage. All cages were retrieved from treated rooms immediately after the exposure duration. Mosquitoes from the individual cages were then transferred into a clean labelled paper cup and provided with 10% sucrose solution on cotton wool. Knocked-down mosquitoes were recorded immediately after transfer, and mortality was recorded at 24 h post-treatment.

Three biological replicates were conducted for *Ae. aegypti* and *Ae. albopictus*, while two biological replicates were performed for *Cx. quinquefasciatus* due to insufficient mosquitoes and logistical constraints. Each replicate was carried out on different occasions, with at least a fortnight between repetitions.

### 2.5. Statistical Analysis

All data were collated and analysed using RStudio (Version 1.3.959, RStudio: Integrated Development for R. RStudio, PBC, Boston, MA, USA). Shapiro–Wilk tests showed that the data did not conform to conditions of normality; hence, non-parametric analyses were used. The Wilcoxon Rank Sum Test was used to compare mortality outcomes between field and susceptible strains of the three mosquito species and between exposed and hidden cages. Additionally, the Kruskal–Wallis Rank Sum Test was used to assess the differences among the three mosquito species. These non-parametric statistical methods were selected to robustly capture variations between the tested groups.

## 3. Results

In our study, each mesh cage contained 15 to 33 female mosquitoes. Across all replicates, Mirakn^®^ GX achieved 100% knockdown, and 99.79 to 100% mortality was observed against field and susceptible strains of *Ae. aegypti*, *Ae. albopictus* and *Cx. quinquefasciatus* (Table 1). Only 3 out of 1508 (0.21%) members of the field strain of *Ae. aegypti* survived exposure, corresponding to a mortality rate of 99.79%. Statistical analysis showed no significant differences between field and susceptible strains of *Ae. aegypti* (*p* = 0.0825), between hidden and exposed cages (*p* = 0.565) or among the three mosquito species (*p* = 0.0787).

## 4. Discussion

Chemical interventions have long been the cornerstone of mosquito vector control during outbreaks aimed at breaking transmission chains [37,38]. However, mosquito vectors have gradually developed resistance mechanisms that enable them to counteract the toxic effects of insecticides [39,40]. In Singapore, field strains of *Ae. aegypti* have exhibited varying levels of pyrethroid resistance in WHO tube bioassays [14,16]. While such bioassays provide evidence of resistance in mosquito populations, they do not directly demonstrate a loss of efficacy under operational conditions. Field evaluations are therefore essential to validate laboratory findings and assess the practical implications of resistance for vector control [41].

Despite the presence of pyrethroid resistance in local *Ae. aegypti* populations, field-collected mosquitoes do not uniformly express resistance traits [14,15]. An important limitation of this study is that insecticide susceptibility testing was not performed on the field-collected mosquito populations used. Although pyrethroid resistance has been reported previously in Singapore’s populations of *Ae. aegypti* and *Ae. albopictus*, the resistance status of the specific mosquito cohorts used here remains unknown. Nonetheless, pyrethroid-formulated products remain efficacious if applied at sufficient doses or having the appropriate formulation characteristics. A laboratory study using WHO intensity bioassays on resistant *Ae. aegypti* has shown that higher deltamethrin concentrations up to 10× the standard dose can still achieve high mortality (98.95%), suggesting that increased concentrations may help overcome resistance and maintain efficacy [40]. However, because susceptibility testing was not performed on the field-collected mosquitoes used in this study, it remains unclear whether the observed efficacy reflects elevated application rates, formulation characteristics, or the inherent susceptibility of the tested population. In the case of Mirakn^®^ GX, although the diagnostic concentration for d,d-trans-cyphenothrin has not been established, its concentration of 0.6% *w*/*w* is comparable to the diagnostic dose of 0.707% *w*/*w* reported for cyphenothrin [42]. However, d,d-trans-cyphenothrin is a chemically purified isomer containing about 98% of the biologically active form [43] compared to cyphenothrin’s 50%. This means that the concentration of d,d-trans-cyphenothrin represents a higher effective dose compared to cyphenothrin. According to the FAO/WHO Evaluation report [26], d,d-trans-cyphenothrin has approximately two times the insecticidal activity of cyphenothrin when tested against the common house mosquito, housefly and German cockroach.

The formulation showed strong adulticidal efficacy (100% knockdown and 99.79% mortality) against both field and susceptible strains of *Ae. aegypti*, *Ae. albopictus* and *Cx. quinquefasciatus*, indicating high effectiveness under the semi-field conditions evaluated. However, the relative contributions of insecticide concentration, formulation characteristics, and mosquito susceptibility could not be determined. The insecticide also exhibited strong adulticidal efficacy, with 99.12 to 100% mortality observed even within hidden areas of the experimental set-up, such as those placed under the bed or behind the curtain. This demonstrates the formulation’s high diffusion capacity and its ability to penetrate potential hidden mosquito resting sites. The observed efficacy could be attributed to the CO_2_ in the formulation, which is known to enhance insecticide dispersion and improve coverage. Previous studies have demonstrated that CO_2_ acts as a highly effective carrier, enhancing the dispersion of insecticides into “hard-to-reach” areas and increasing overall treatment efficacy against various stored-product pests [17,19]. These findings suggest that the CO_2_-based formulation facilitates superior insecticide penetration, increasing the likelihood of direct contact with mosquitoes even in hidden resting sites, thus maximising its adulticidal activity.

Beyond enhancing the dispersion of insecticide, CO_2_ can influence mosquito physiological processes. Studies have reported that CO_2_ can enhance insecticide toxicity by prolonging or irreversibly opening spiracles, resulting in increased uptakes of insecticides [37,38,39,40]. In cockroaches, spiracles serve as a primary penetration route for aerosolised pyrethroids, directly affecting knockdown efficacy [44]. Similar studies on stored-product pests showed that spiracles are also a major entry point for fumigants or aerosolised insecticides, demonstrating that CO_2_-driven spiracular responses can further enhance mortality [17,19,45,46]. Since *Ae. aegypti* are also reported to undergo extensive spiracular opening even in the presence of small concentrations of CO_2_ [47], a similar enhancement of insecticide uptake in the presence of CO_2_ by the study’s test mosquitoes is also likely. While these observations suggest plausible mechanisms through which CO_2_ may enhance insecticide delivery or mosquito exposure, the present study was not designed to evaluate the independent contribution of CO_2_, as a CO_2_-only control formulation was not available during the study period. Thus, our interpretation remains a hypothesis derived from these parallel systems. Consequently, the observed efficacy should be attributed to the complete formulation rather than to any specific contribution of CO_2_. Future work is necessary to isolate and quantify any direct or synergistic effects of CO_2_ on mosquitoes.

This study also explored operational practicality, where a single experimental trial was conducted in Room 1 and Room 2, with the exposure duration reduced to 10 min. Additional mosquito cages were strategically placed throughout the premises to simulate typical mosquito resting sites (as depicted in Supplementary Figure S1). Reducing the exposure duration from 60 to 10 min still achieved full knockdown and mortality against both field and susceptible strains of *Ae. aegypti* (as shown in Supplementary Table S1), suggesting that 10 min exposure duration is sufficient. A shortened exposure duration could minimise disruption to occupants and increase community acceptance of indoor space spraying operations.

As summarised in Supplementary Table S2, the insecticide product offers several practical advantages over conventional indoor ULV spraying. It is odourless, leaves no wet residues or solvent stains on surfaces, and does not require an external power source, reducing operational complexity and potential hazards associated with electrical equipment. Furthermore, the high diffusion capability of the formulation may improve the coverage of hidden or hard-to-reach resting sites, as observed by the high efficacy against *Ae. aegypti*, *Ae. albopictus*, and *Cx. quinquefasciatus.* This also reduces the treatment time and the time required for occupants to vacate the premises. Based on supplier-derived estimates, which incorporate differences in operational cost (per number of units and associated manpower requirements), the main drawback of Mirakn^®^ GX is its relatively higher overall cost compared to conventional indoor ULV spraying.

Importantly, no occupants reported being unwell throughout the study period. These findings align with feedback from residents during the preliminary Mirakn^®^ GX acceptance trial conducted by NEA at the Serangoon Gardens Estate, where 18 households across two streets participated and consented to this novel treatment approach.

While this study provides valuable insights into the use of Mirakn^®^ GX as an alternative for indoor ULV spraying, the additional limitations need to be acknowledged. (1) These include the unavailability of a compressed gas tank without the active ingredient, which restricted the investigation of the isolated effect of CO_2_, a potential area for future research. (2) This study was conducted in a single occupied residential apartment unit comprising two rooms, representative of a typical public housing household in Singapore. While this provides a realistic operational setting, further evaluation across a wider range of housing layouts, room sizes, furnishing densities, ventilation characteristics, and housing types would help to strengthen the generalisability of the findings and provide additional support for the use of this insecticide in indoor space spraying. (3) Only two biological replicates were available for *Cx. quinquefasciatus* because of mosquito supply constraints. Although mortality was consistently 100% across both replicates, additional replication would improve confidence in the observed efficacy.

The present study followed WHO efficacy-testing guidelines using caged sentinel mosquitoes positioned at exposed and hidden locations. This methodology provides a standardised and reproducible assessment of insecticidal efficacy and facilitates comparison across studies. Although free-flying mosquito evaluations may offer additional insights into operational performance under real-world conditions, they are associated with greater variability in mosquito movement, exposure, and recapture. Future studies incorporating free-flying mosquitoes may be considered to complement the present findings and contribute additional evidence on operational effectiveness.

The growing problem of pyrethroid resistance in *Ae. aegypti*, *Ae. albopictus* and *Cx. quinquefasciatus* has been well documented globally [40,48,49,50,51], highlighting the need for the judicious use of insecticides, rotation between different insecticide classes, and adoption of integrated vector management (IVM) strategies to slow resistance development. Although pyrethroid resistance has previously been documented in Singapore’s *Ae. aegypti* populations, the susceptibility status of the field-collected mosquitoes evaluated in this study was not determined. Therefore, the present findings demonstrate efficacy against locally collected mosquito populations under the conditions tested but do not allow for conclusions regarding the efficacy against resistance-characterised populations.

## Figures and Tables

**Table 1:** High mean % knockdown ^^^ and mean % mortality ^#^ observed in field and susceptible strains of *Ae. aegypti*, *Ae. albopictus* and *Cx. quinquefasciatus* indicate the strong effectiveness of Mirakn^®^ GX.

Test Species	Mosquito Strain	Room	Number of Mosquitoes Tested	Mean % Knockdown ^^^	Mean % Mortality ^#^ ± SE
*Ae. aegypti*	Field		1508	100.00	99.79 ± 0.12
		1	774	100.00	100.00
			Exposed: 598	100.00	100.00
			Hidden: 176	100.00	100.00
		2	734	100.00	99.56 ± 0.25
			Exposed: 594	100.00	99.65 ± 0.24
			Hidden: 140	100.00	99.12 ± 0.88
	Susceptible (Bora-Bora)		1525	100.00	100.00
		1	787	100.00	100.00
			Exposed: 599	100.00	100.00
			Hidden: 188	100.00	100.00
		2	738	100.00	100.00
			Exposed: 612	100.00	100.00
			Hidden: 126	100.00	100.00
*Ae. albopictus*	Field		1408	100.00	100.00
		1	737	100.00	100.00
			Exposed: 553	100.00	100.00
			Hidden: 184	100.00	100.00
		2	671	100.00	100.00
			Exposed: 551	100.00	100.00
			Hidden: 120	100.00	100.00
	Susceptible (NEA-EHI strain)		1549	100.00	100.00
		1	787	100.00	100.00
			Exposed: 586	100.00	100.00
			Hidden: 201	100.00	100.00
		2	762	100.00	100.00
			Exposed: 627	100.00	100.00
			Hidden: 135	100.00	100.00
*Cx. quinquefasciatus*	Field		977	100.00	100.00
		1	537	100.00	100.00
			Exposed: 407	100.00	100.00
			Hidden: 130	100.00	100.00
		2	440	100.00	100.00
			Exposed: 359	100.00	100.00
			Hidden: 81	100.00	100.00
	Susceptible (NEA-EHI strain)		1003	100.00	100.00
		1	529	100.00	100.00
			Exposed: 401	100.00	100.00
			Hidden: 128	100.00	100.00
		2	474	100.00	100.00
			Exposed: 395	100.00	100.00
			Hidden: 79	100.00	100.00

^^^ Knockdown was observed 60 min after treatment. ^#^ Mortality was observed 24 h after treatment.

## Data Availability

The datasets generated during and/or analysed during the current study are available from the corresponding author on reasonable request.

## Supplementary Materials

The following supporting information can be downloaded at https://www.mdpi.com/article/10.3390/insects17070716/s1, Figure S1: Schematic diagram of the residential apartment unit showing mesh cage positions for the “adjusted exposure duration” efficacy test; Table S1: Mean % knockdown ^^^ and total % mortality ^#^ for field and susceptible strains of *Ae. aegypti* show the high effectiveness of Mirakn^®^ GX; Table S2: Advantages and disadvantages between Mirakn^®^ GX and conventional indoor ULV spraying.
