# Supplementary material for: A Unique Desiccated Carbon Dioxide (CO2) Formulation of d,d-trans-Cyphenothrin, Mirakn® GX: A Novel Alternative to Indoor Ultra-Low-Volume Spraying
Source: Insects. 2026 Jul 10;17(7):716. doi: 10.3390/insects17070716 (PMC13411954; doi:10.3390/insects17070716)
Supplement: Supplementary file 1 [file insects-17-00716-s001.zip › insects-4306881-supplementary.pdf]

SUPPLEMENTARY INFORMATION

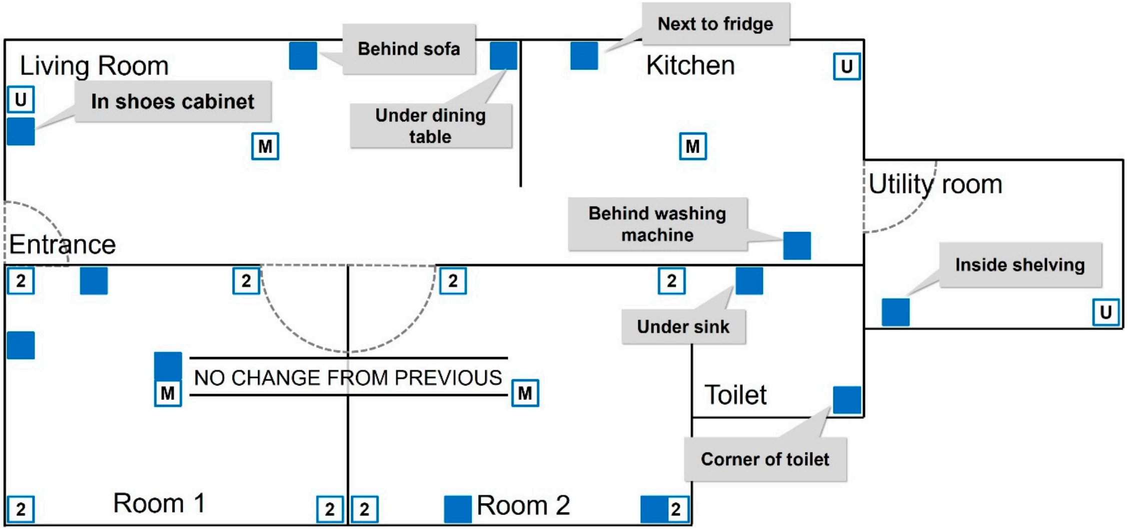

Supplementary Figure S1 Schematic diagram of the residential apartment unit showing mesh cage positions for the "adjusted exposure duration" efficacy test. At each mesh cage position, one cage of field and one of susceptible strains mosquitoes are placed. Non-filled and filled squares represent exposed and hidden cages, respectively. "2" indicates cages placed 0.25 m below the ceiling and 0.25 m above the floor and "M" indicates cages placed mid-height (1.5 m) at the centre of the room. "U" indicates cages placed at upper most window grille. The dashed arc indicates the door swing path.

Supplementary Table S1. Mean % knockdown^ and total % mortality# for field and susceptible strains of *Ae. aegypti* show the high effectiveness of Mirakn® GX.

Test species	Strain	Number of mosquitoes tested	Mean % knockdown^	Mean % mortality#
Ae. aegypti	Field	884<br>Exposed: 638<br>Hidden: 246	100.00	100.00
	Susceptible (Bora-Bora)	913<br>Exposed: 668<br>Hidden: 245	100.00	100.00

^ Knockdown was observed 60 min after treatment

Mortality was observed 24 h after treatment

Supplementary Table S2. Advantages and disadvantages between Mirakn® GX and conventional indoor ULV spraying

	**Mirakn® GX**	**Conventional indoor ULV spraying**
Advantages	▪ Ready-to-use<br>▪ Non-flammable<br>▪ High diffusion<br>▪ Odourless<br>▪ No wet residues on surfaces<br>▪ No external power source needed<br>▪ Shorter treatment time	▪ Ready-to-use<br>▪ Non-flammable<br>▪ Lower overall cost
Disadvantages	▪ Higher overall cost	▪ Cumbersome<br>▪ Electrically operated

		▪ Potentially trip hazard and electrical hazard risk for those required external power<br>▪ Wet surface residues<br>▪ Longer treatment time
